# Outpatient Cutaneous Wound Care in the United States: Specialty Distribution and Antimicrobial Prescribing Patterns

**DOI:** 10.3390/antibiotics15020142

**Published:** 2026-02-01

**Authors:** Ayman Grada, Rithi John Chandy, Jiwon Park, Steven R. Feldman

**Affiliations:** 1 Department of Dermatology, Case Western Reserve University School of Medicine, Cleveland, OH 44106, USA; 2 Center for Dermatology Research, Department of Dermatology, Wake Forest School of Medicine, Winston-Salem, NC 27104, USA; 3 Long School of Medicine, UT Health San Antonio, San Antonio, TX 78229, USA

**Keywords:** antibiotics, antimicrobial stewardship, wound infection, cutaneous wounds, chronic wounds, primary care, dermatology, cephalexin, topical antimicrobials, NAMCS

## Abstract

**Background:** Cutaneous wounds are common in outpatient care, but national patterns of who manages them and how antimicrobials are used remain unclear. **Objectives**: To characterize outpatient specialty involvement and antimicrobial use for acute and chronic cutaneous wound visits in the United States. **Methods**: We conducted a retrospective cross-sectional analysis of 2011–2019 National Ambulatory Medical Care Survey (NAMCS) data. Cutaneous wound visits were identified using prespecified ICD-9-CM and ICD-10-CM codes and classified as acute (open or traumatic wounds and burns) or chronic (pressure injuries and lower-limb ulcers). Survey weights were applied to estimate national visit volumes, specialty shares, and antimicrobial utilization patterns. **Results**: We identified 45.1 million cutaneous wound visits, representing 0.8% of all outpatient visits, of which about two thirds were acute and one third chronic. Primary care physicians accounted for the largest share of wound visits, while dermatologists managed 3.9% of overall wound visits, 2.4% of acute visits, and 7.4% of chronic visits. Among 156.6 million medications recorded at wound visits, antimicrobials represented 13.1% overall, 14.9% in acute visits, and 10.2% in chronic visits. Cephalexin accounted for 32.1% of antimicrobial medications overall and 39.2% in acute visits, whereas chronic wound visits had a more heterogeneous antimicrobial profile that included topical mupirocin, cephalexin, trimethoprim–sulfamethoxazole, and topical nystatin. **Conclusions**: Outpatient cutaneous wound care in the United States is delivered predominantly by primary care clinicians and relies heavily on a small set of systemic and topical antimicrobials, highlighting opportunities to strengthen antimicrobial stewardship and expand dermatology’s role in chronic wound management.

## 1. Introduction

Cutaneous wounds, including acute traumatic injuries and chronic ulcers, are common in outpatient practice and contribute substantially to morbidity, disability, and health care expenditures in the United States [1,2]. Chronic nonhealing wounds such as venous leg ulcers, diabetic foot ulcers, and pressure injuries affect millions of adults and are associated with impaired quality of life, limb loss, and premature mortality [1,3]. Recent economic evaluations estimate that chronic wounds impose tens of billions of dollars in annual costs on the US health care system, largely through outpatient visits, hospitalizations, procedures, and long-term supportive care [4,5]. Clinically, chronic wounds are often described as wounds that fail to progress through an orderly and timely reparative process, commonly operationalized as limited healing progress over approximately 4 to 6 weeks [6,7].

Best practice guidance for wound management emphasizes meticulous local care, pressure relief, optimization of perfusion and glycemic control, and timely debridement, with systemic antibiotics reserved for wounds that show clear clinical signs of infection and are tailored to likely pathogens [8,9,10,11]. Routine use of systemic antibiotics for clinically uninfected or colonized wounds is discouraged because it offers little benefit and can promote antimicrobial resistance, drug-related adverse events, and Clostridioides difficile infection [9,12]. Topical antimicrobials and antiseptics may have a role in selected situations but should also be used judiciously as part of a broader wound hygiene and stewardship strategy [13,14,15,16].

Despite these recommendations, contemporary national patterns of antimicrobial use for cutaneous wounds in routine outpatient care remain incompletely characterized. It is not well-known which clinician specialties most commonly manage acute and chronic cutaneous wounds in office-based settings, how frequently antimicrobials are used during these visits, and which specific systemic and topical agents dominate prescribing. Understanding these patterns is important to identify high-yield targets for outpatient antimicrobial stewardship and to clarify the potential role of dermatology relative to primary care and surgical specialties.

The National Ambulatory Medical Care Survey (NAMCS) is a long-standing, nationally representative survey of office-based physician visits in the United States conducted by the National Center for Health Statistics using a multistage probability design [17,18]. NAMCS captures patient diagnoses, physician specialty, and medications recorded at each sampled visit, which makes it well suited to describe national patterns of cutaneous wound care and associated antimicrobial use.

In this study, we used NAMCS data from 2011 to 2019 to provide a comprehensive description of outpatient cutaneous wound care in the United States. Our objectives were to: (1) estimate the national volume of acute and chronic cutaneous wound visits; (2) describe the specialty distribution of these visits; and (3) characterize antimicrobial prescribing patterns, including the relative use of systemic and topical agents and the most frequently used antimicrobial medications, overall and by wound type. We discuss these patterns in the context of antimicrobial stewardship and potential opportunities for enhanced dermatology involvement in wound care.

## 2. Results

### 2.1. Volume and Diagnostic Mix of Cutaneous Wound Visits

Across NAMCS years 2011–2019, an estimated 5.76 billion office-based outpatient visits occurred in the United States. Of these, 45.1 million visits (0.78%) had at least one diagnosis code corresponding to a cutaneous wound. Approximately 33 million visits involved acute wounds, and 13 million involved chronic wounds. Acute wounds therefore accounted for about two thirds of cutaneous wound visits, with chronic wounds comprising the remaining one third.

The diagnostic mix was concentrated in a relatively small number of codes. The top twenty cutaneous wound diagnoses accounted for 62.7% of all wound diagnoses, 71.9% of acute wound diagnoses, and 89.5% of chronic wound diagnoses (Supplementary Table S1). Acute wound visits were dominated by traumatic open wounds of the extremities, particularly open wounds of the thumb, lower leg, ear, fingers, and foot. Chronic wound visits were driven by non-pressure ulcers of the foot and lower limb and by pressure injuries, with the top five chronic diagnoses together accounting for 49.7% of chronic wound diagnoses.

In trend analyses of visit volumes, annual frequencies of overall, acute, and chronic cutaneous wound visits did not show statistically significant linear changes over time (all *p* ≥ 0.05), indicating that the outpatient burden of cutaneous wound visits remained relatively stable from 2011 to 2019.

### 2.2. Specialty Distribution of Wound Visits

Detailed physician specialty information was available in NAMCS public-use files for 2011 and 2013–2016. In these years, primary care alone accounted for about half of cutaneous wound visits, and together with surgical specialties represented the majority of wound care. For overall cutaneous wound visits, general or family practice represented 27.8%, internal medicine 16.0%, pediatrics 9.5%, orthopedic surgery 9.9%, and general surgery 3.9%, with “other specialties” contributing 25.4% (Table 1). “Other specialties” aggregates office-based physician subspecialties not listed separately in NAMCS (for example, rheumatology, endocrinology, gastroenterology, nephrology, pulmonology, allergy and immunology, geriatrics, and physical medicine and rehabilitation). Dermatology managed 3.85% of all cutaneous wound visits, while otolaryngology, obstetrics and gynecology, cardiovascular diseases, and other subspecialties each accounted for smaller fractions.

Dermatology’s contribution differed by wound type. Dermatologists managed an estimated 2.35% of acute wound visits and 7.39% of chronic wound visits, indicating a relatively larger role in chronic wound care despite a small share of overall wound visits.

**Figure 1:**
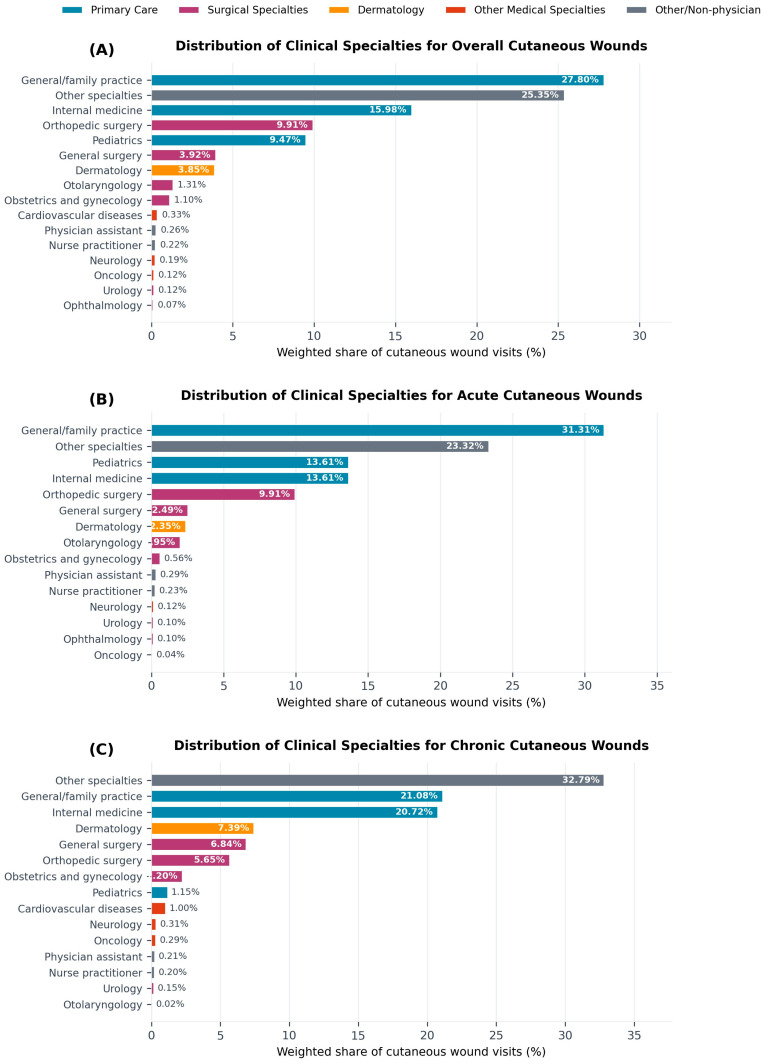
Weighted share of cutaneous wound visits by clinical specialty for (**A**) overall wounds, (**B**) acute wounds, and (**C**) chronic wounds, NAMCS 2011 and 2013–2016. Specialties are color-coded by category: primary care (blue), surgical specialties (magenta), dermatology (orange), other medical specialties (red), and other/non-physician providers (gray).

To align with the broader NAMCS physician category variable, which groups clinicians into primary care, surgical specialties, and medical specialties modeled on American Medical Association categories, we further collapsed individual specialties into these three groups. In this classification, general or family practice, internal medicine, and pediatrics are grouped as primary care; orthopedic surgery, general surgery, otolaryngology, obstetrics and gynecology, urology, and related procedural fields are grouped as surgical specialties; and dermatology along with other internal medicine subspecialties are grouped as medical specialties. Using these categories, primary care clinicians were the most frequent providers of cutaneous wound care (Table 2). Primary care accounted for 43.5% of acute wound visits, 51.3% of chronic wound visits, and 46.1% of all cutaneous wound visits. Surgical specialties accounted for 24.1% of acute, 29.9% of chronic, and 25.7% of overall wound visits, while medical specialties (including dermatology) accounted for 32.4%, 18.7%, and 28.1%, respectively. Figure 1 illustrates the distribution of cutaneous wound visits by specialty and by wound type.

### 2.3. Antimicrobial Use Among Medications at Wound Visits

Across all cutaneous wound visits, clinicians recorded an estimated 156.6 million medications. Of these, antimicrobials (antibiotics, antifungals, and antivirals) represented 13.1% of all medications at wound visits, with antimicrobials accounting for 14.9% of medications in acute wound visits and 10.2% in chronic wound visits (Table 3). This corresponded to approximately 20.5 million antimicrobial medications recorded at cutaneous wound visits over the study period. Most medications used at wound visits were prescription products. Overall, 73.1% of medications were prescription-only, 22.1% could be provided as either prescription or over-the-counter, and only 2.2% were nonprescription drugs, a pattern that was similar for acute and chronic wounds (Supplementary Table S2). Thus, more than 95% of medications used in wound visits were prescription-capable, suggesting that stewardship efforts in cutaneous wound care will primarily involve prescribed therapies.

Among antimicrobial medications, systemic agents comprised 52.8% overall, including 60.5% of antimicrobials in acute wound visits and 42.6% in chronic wound visits, with the remainder being topical agents (values derived from NAMCS-weighted antimicrobial counts; Supplementary Figure S2).

### 2.4. Composition of Specific Antimicrobial Agents

Antimicrobial use during cutaneous wound visits relied heavily on a small number of core agents (Table 3; Supplementary Figure S1). Using antimicrobial medications as the denominator, the first-generation cephalosporin cephalexin was the most commonly used agent overall, accounting for 32.1% of all antimicrobial medications and 4.22% of all medications recorded at cutaneous wound visits. Topical mupirocin was the second most frequently used antimicrobial (12.1% of antimicrobial medications; 1.59% of all medications), followed by a grouped “miscellaneous antibiotic” category.

In acute wound visits, cephalexin was even more dominant, representing 39.2% of antimicrobial medications and 5.84% of all medications, with topical mupirocin and silver sulfadiazine also commonly used (Table 3). In chronic wound visits, the antimicrobial profile was more heterogeneous. Topical mupirocin (14.0% of antimicrobial medications), cephalexin (11.9%), trimethoprim–sulfamethoxazole (10.0%), topical nystatin (7.4%), topical chlorhexidine (6.3%), and fluconazole (4.8%) were among the most frequently used agents, reflecting a mix of systemic antibiotics, topical antibacterials, and topical antifungals (Table 3; Supplementary Figure S3).

**Figure 2:**
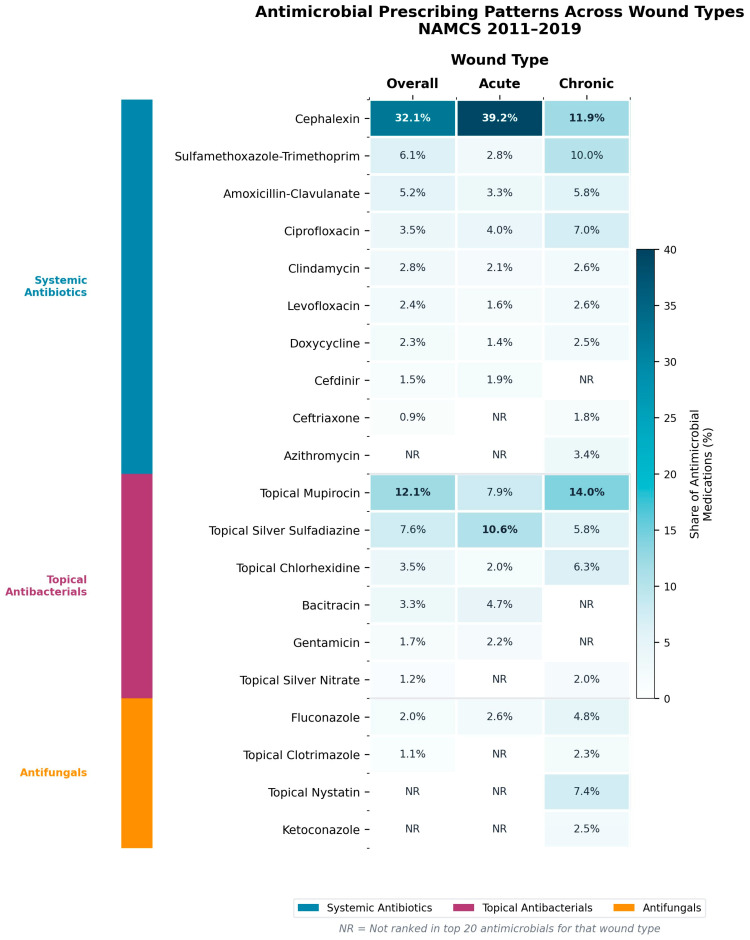
Heatmap of the top antimicrobial agents at cutaneous wound visits overall and by wound type, NAMCS 2011–2019. Antimicrobials are grouped by class: systemic antibiotics, topical antibacterials, and antifungals. Values represent each agent’s share of all antimicrobial medications recorded at wound visits. NR = not ranked in the top 20 antimicrobials for that wound type.

Across all wound visits, the top three antimicrobial agents (cephalexin, topical mupirocin, and miscellaneous antibiotics) accounted for 53.3% of all antimicrobial medications, the top five agents for 67.0%, and the top ten for 85.3%. Concentration was strongest in acute wounds, where the top three agents accounted for 60.7% and the top ten for 88.0% of antimicrobial medications. In chronic wounds, the top three and top five agents represented 35.9% and 50.4% of antimicrobial medications, respectively, and the top ten accounted for 76.5%, consistent with a more diffuse antimicrobial profile (Table 3). These patterns are illustrated in Figure 2, which presents a heatmap of the most commonly used antimicrobial agents overall and stratified by wound type.

### 2.5. Time Trends in Antimicrobials and Specialty Mix

In trend analyses, the proportions of antimicrobials, antibiotics, and antifungals among medications at cutaneous wound visits did not show statistically significant linear changes over time (all *p* ≥ 0.09). The only significant temporal pattern was a modest increase in the proportion of antiviral agents among antimicrobial medications in chronic wound visits (*p* = 0.037), while antiviral use in acute wound visits remained low and stable (Supplementary Table S3).

Similarly, there were no statistically significant linear trends in the relative contributions of primary care, surgical, or medical specialties to acute, chronic, or overall cutaneous wound visits during the study period (all *p* > 0.05; Supplementary Table S4). The specialty mix of outpatient cutaneous wound care therefore appeared stable over time.

## 3. Discussion

In this nationally representative analysis of US office-based visits, cutaneous wound care occupied a small but stable share of ambulatory practice, with approximately one in 120 visits involving an acute or chronic cutaneous wound. Acute wounds accounted for about two thirds of visits and were dominated by traumatic open injuries of the extremities, whereas chronic wounds comprised about one third and were largely lower-extremity ulcers and pressure injuries. These patterns are consistent with prior work showing that chronic wounds, particularly lower-limb ulcers, account for a disproportionate share of morbidity and costs despite representing a minority of encounters [4,19,20].

Antimicrobial use during wound visits was substantial and concentrated in a limited repertoire of agents. Across all wound visits, more than one in eight medications was an antimicrobial, and just a few drugs accounted for most antimicrobial use. Cephalexin alone represented roughly one third of antimicrobial medications overall and nearly 40% in acute wound visits, while topical mupirocin was the second most frequently used agent. Cephalexin dominates acute wound prescribing because it is an oral, inexpensive, first-generation cephalosporin with good activity against common Gram-positive skin pathogens, is widely recommended as a first-line oral option for uncomplicated nonpurulent cellulitis and other skin and soft tissue infections and is deeply familiar to outpatient prescribers. In chronic wound visits, antimicrobial selection was more heterogeneous but still centered on a small group of systemic antibiotics and topical antibacterials and antifungals.

Antimicrobial stewardship and wound care guidelines emphasize that systemic antibiotics should be reserved for clinically infected wounds and that colonized or noninfected wounds should be managed with local wound care, debridement, offloading, and optimized perfusion rather than routine systemic therapy [9,11,21,22,23]. In outpatient practice, features suggestive of localized wound infection may include increasing erythema, warmth, swelling, tenderness, purulent drainage, malodor, or worsening wound appearance, whereas systemic infection may be suggested by fever or other systemic symptoms [24,25]. The predominance of systemic agents among antimicrobials in our analysis, particularly for acute wounds, may be appropriate in many high-risk traumatic injuries but also suggests persistent opportunities for stewardship. In the absence of clinical details regarding infection status, culture results, or systemic signs, we cannot determine the appropriateness of individual prescriptions. However, the stability of antimicrobial shares over nearly a decade, despite increasing attention to antimicrobial stewardship in wound care, supports the need for targeted outpatient interventions. These might include decision support to distinguish infection from colonization, clearer outpatient criteria for when systemic therapy adds value beyond local care, training and decision support on narrow-spectrum selection and appropriate duration, and focused stewardship guidance for high-frequency agents such as cephalexin and topical mupirocin.

The heavy and sustained use of topical antimicrobials, particularly mupirocin, also warrants attention. Mupirocin is an important agent for decolonization and treatment of localized *Staphylococcus aureus* (*S. aureus*) infections, yet repeated or widespread use has been associated with increased resistance in *S. aureus* and coagulase-negative *staphylococci* [26,27,28]. Our findings that mupirocin is one of the most frequently used agents in both acute and chronic wound care highlight the potential value of wound-specific stewardship guidance that addresses not only systemic antibiotics but also topical agents and antiseptics. In routine wound care, clinicians may also use adjunctive non-medication strategies with antimicrobial properties such as antimicrobial dressings (for example, silver-based dressings) and topical medical-grade honey products [13], which are not reliably captured in NAMCS medication fields. Aligning topical antimicrobial use with guideline-supported indications could help preserve efficacy while minimizing selection pressure for resistance.

In chronic wound care, the more diverse antimicrobial profile that includes topical nystatin, systemic antifungals, and broad-spectrum antibiotics raises questions about how consistently treatment aligns with evidence-based recommendations. Chronic lower-limb ulcers and pressure injuries are frequently colonized by polymicrobial flora rather than overtly infected [29,30,31] and antimicrobial therapy that is not targeted to clearly defined infection may not improve healing while increasing the risks of resistance, drug toxicity, and *Clostridioides difficile* infection. Stewardship efforts in chronic wound care therefore need to address not only the volume of systemic antibiotics but also the indications for antifungal and broad-spectrum antibacterial agents, and to reinforce the central role of local wound care, debridement, and optimization of perfusion and comorbid conditions.

The specialty distribution of wound visits underscores that primary care clinicians and surgical specialties are the principal providers of ambulatory wound care in the United States. Primary care accounted for nearly half of acute, chronic, and overall wound visits, and surgical specialties contributed an additional quarter to one third. Dermatology managed fewer than 4% of overall wound visits but a relatively larger share of chronic wound visits, approaching 7% of chronic encounters. This pattern suggests that dermatologists are more often involved when wounds are complex or refractory, yet they remain responsible for only a small fraction of chronic wound care nationally. Survey data from US dermatology residents indicate that most trainees do not feel adequately prepared to manage acute or chronic wounds and that fewer than half intend to incorporate wound care into their future practice, which suggests that gaps in residency training may contribute to dermatology’s limited role in this area [32]. Other factors, such as competing procedural priorities and financial or organizational incentives, may also play a role, although these have not been systematically studied. Given dermatology’s expertise in skin biology, inflammation, and cutaneous infection, expanding dermatology involvement through multidisciplinary wound clinics, structured referral pathways, and teledermatology consultation could help support more precise diagnosis, biopsy and culture when indicated, and evidence-based antimicrobial selection.

Our results also show that visit volumes, specialty mix, and antimicrobial shares remained statistically stable from 2011 to 2019, with the exception of a modest increase in antiviral use for chronic wounds. The stability of wound visit frequency suggests that the outpatient burden of cutaneous wounds has not diminished over time, despite advances in preventive care and chronic disease management. The absence of major shifts in specialty mix indicates that primary care and surgical specialists have continued to shoulder most wound care responsibilities, without a measurable increase in dermatology’s contribution. Similarly, the lack of clear downward trends in antimicrobial shares implies that outpatient stewardship efforts have not yet translated into detectable reductions in overall antimicrobial use for wounds at the national level. These findings provide a useful pre–COVID-19 benchmark against which future changes in practice, including those driven by evolving stewardship initiatives and post-pandemic care models, can be assessed.

This study has several strengths. We used a large, nationally representative dataset with complex survey weights that captures office-based care across multiple specialties over nearly a decade. We applied consistent case definitions to distinguish acute from chronic wounds and mapped medications to clinically meaningful antimicrobial categories, which allowed us to characterize not only whether antimicrobials were used but also which specific agents and classes predominated. The integration of specialty, diagnosis, and medication information within a single dataset provided a comprehensive view of who delivers cutaneous wound care and how antimicrobials are deployed in routine outpatient practice.

Important limitations should be noted. NAMCS is a visit-based survey, so repeated visits by the same patient cannot be linked, and we cannot evaluate longitudinal outcomes, treatment duration, or time to healing. NAMCS does not include wound duration or time since onset, so temporal chronicity cannot be assessed, and some misclassification of acute versus chronic presentation is possible. Care patterns and antimicrobial selection may differ by patient demographics, insurance status, and geographic region, and residual confounding by these factors is possible in this descriptive, visit-based analysis. The dataset lacks detailed clinical information on wound severity and wound examination findings, microbiologic results, and systemic signs or symptoms of infection, which precludes assessment of whether antimicrobial prescribing was clinically appropriate. NAMCS does not reliably capture dressings, devices, clinic-applied products, or many over-the-counter wound products, limiting inference regarding these non-medication antimicrobial strategies. Diagnosis coding may be incomplete or heterogeneous, especially for specific chronic wound subtypes such as diabetic foot ulcers, and some complex wounds may be underrepresented if they are preferentially managed in hospital-based or non-office settings, which may also contribute to underrepresentation of services such as plastic surgery. Specialty information is available only for selected years and is aggregated into broad categories that may mask variation within specialties. Accordingly, agent-level antimicrobial selection stratified by detailed specialty and wound type is constrained in NAMCS public-use files because stratification by wound type, specialty, and specific agent can yield sparse cells under NAMCS reliability guidance. Finally, our estimates describe the share of antimicrobial medications among all medications recorded, not the proportion of visits in which at least one antimicrobial was prescribed, and therefore should be interpreted as patterns of drug selection rather than visit-level prescribing rates.

Despite these limitations, this analysis provides a national overview of outpatient cutaneous wound care in the pre-COVID-19 era and highlights several opportunities to improve practice. For antimicrobial stewardship, priorities include refining criteria for systemic therapy, encouraging judicious use of high-volume agents such as cephalexin and mupirocin, and developing wound-focused stewardship tools that are usable in primary care and surgical settings where most wound care occurs. For specialty allocation, there is a rationale for increasing dermatology’s participation in chronic wound management through collaborative care models that integrate dermatologists with primary care, surgery, vascular medicine, podiatry, and infectious diseases. Future research should examine how specific stewardship interventions and multidisciplinary models influence antimicrobial use, resistance patterns, and patient-centered outcomes in both acute and chronic cutaneous wounds. In addition, studies using claims, electronic health records, or wound registries with richer clinical detail and stable specialty capture should evaluate specialty-specific antimicrobial selection and appropriateness, including infection indicators and wound characteristics.

## 4. Materials and Methods

### 4.1. Data Source and Study Design

We performed a cross-sectional analysis of the National Ambulatory Medical Care Survey (NAMCS) public use files from 2011 to 2019. NAMCS is an annual, nationally representative survey of visits to non-federal, office-based physicians in the United States conducted by the National Center for Health Statistics using a multistage probability design with visit weights, strata, and primary sampling units. NAMCS samples non-federal, office-based physicians; visits to hospital-based outpatient clinics, emergency departments, and settings where nonphysician clinicians are the primary providers are not captured in this survey and were therefore outside the scope of our analysis.

### 4.2. Case Definitions for Cutaneous Wounds

Cutaneous wound visits were identified using all available diagnosis fields. International Classification of Diseases, Ninth Revision, Clinical Modification (ICD 9 CM) codes were used before October 2015 and International Classification of Diseases, Tenth Revision, Clinical Modification (ICD 10 CM) codes thereafter.

We defined cutaneous wounds as diagnoses representing an acute or chronic break in skin integrity. Acute wounds included traumatic open or complex wounds and thermal injuries of the skin and extremities. Chronic wounds included pressure injuries and non-pressure lower extremity ulcers such as venous, arterial, and diabetic ulcers. Visits with at least one acute wound code were classified as acute. Visits with at least one chronic wound code were classified as chronic. Because NAMCS does not capture wound duration or time since onset, we classified acute and chronic wounds using diagnosis-code groupings rather than temporal definitions of chronicity. ICD-9-CM and ICD-10-CM code lists were constructed and mapped across coding eras, but completeness cannot be guaranteed and some diagnostic misclassification is possible. The full code lists are provided in Supplementary Methods Table S1.

### 4.3. Medications and Antimicrobial Classification

NAMCS records medications prescribed, provided, or continued at each visit (up to eight medications per visit in 2011–2013 and up to thirty thereafter). All medications recorded at cutaneous wound visits were abstracted. Antimicrobial medications were defined as systemic or topical antibiotics, antifungals, or antivirals based on NAMCS therapeutic classes and generic names. Topical antiseptics, dressings, advanced wound products, biologic agents, and other non-antimicrobial therapies were not counted as antimicrobials. Because these non-medication wound products are not reliably captured in NAMCS medication fields, our antimicrobial measures reflect medication mentions only and do not quantify national use of antimicrobial dressings or related wound-care products. Antimicrobials were classified by route (systemic or topical) and by class (antibiotic, antifungal, antiviral). Mapping details are provided in Supplementary Methods Table S2.

Medication analyses used individual drug mentions as the unit of analysis and described the proportion of antimicrobial medications among all medications recorded at wound visits.

### 4.4. Clinician Specialty and Provider Categories

Physician specialty was described using both detailed specialty codes (available for 2011 and 2013–2016) and a broader NAMCS physician category variable that groups specialties as primary care, surgical specialties, or medical specialties. The NAMCS physician category framework is modeled on American Medical Association specialty groupings, supporting cross-year consistency and cross-study comparability. In the public-use NAMCS files, detailed specialty categories are not consistently available across years, and some specialties may be aggregated; accordingly, certain specialties, including plastic surgery, may not be separately identifiable and may be incorporated within broader categories depending on the survey year. General or family practice, internal medicine, and pediatrics were classified as primary care. Orthopedic surgery, general surgery, otolaryngology, obstetrics and gynecology, urology, and related fields were classified as surgical specialties. Dermatology and other internal medicine subspecialties were classified as medical specialties. Dermatology-specific estimates were also reported when detailed codes were available. The specialty mapping is shown in Supplementary Methods Table S3.

### 4.5. Outcomes and Statistical Analysis

Primary outcomes were: (1) nationally weighted counts and proportions of acute, chronic, and overall cutaneous wound visits among all office-based physician visits; (2) the distribution of wound visits across specialties; and (3) the proportion and composition of antimicrobial medications at wound visits. Survey procedures that incorporate NAMCS visit weights, strata, and primary sampling units were used to generate national estimates. All weighted counts represent survey-weighted national estimates of visits or medication mentions. Linear trends over time were evaluated by fitting survey-weighted linear regression models with calendar year as a continuous predictor. Two-sided *p* values for trend were obtained from the coefficient for calendar year, and *p* < 0.05 was considered statistically significant. Following National Center for Health Statistics guidance, estimates based on fewer than 30 unweighted observations or with a relative standard error greater than thirty percent were considered unreliable and were suppressed or combined. All analyses were conducted using SAS version 9.4 (SAS Institute, Cary, NC, USA). NAMCS public use files contain de-identified data, and secondary analyses are considered exempt from additional institutional review board review.

## 5. Conclusions

In this nationally representative analysis, cutaneous wound visits accounted for a small but stable share of US office-based care and were managed largely in primary care and surgical settings. Antimicrobials made up a substantial proportion of medications at these visits and were dominated by a few systemic and topical agents, with little evidence of change over time. These patterns highlight opportunities to strengthen outpatient antimicrobial stewardship for acute and chronic wounds and to expand dermatology’s role within multidisciplinary wound care models, and they provide a pre-COVID-19 benchmark against which the impact of future stewardship and care delivery initiatives can be assessed.

## Figures and Tables

**Table 1:** Specialty of physicians managing cutaneous wound visits in US office-based practice, NAMCS 2011 and 2013–2016.

Overall Cutaneous Wounds								
Rank	Specialty	2011, Weighted Visits, *n*	2013, Weighted Visits, *n*	2014, Weighted Visits, *n*	2015, Weighted Visits, *n*	2016, Weighted Visits, *n*	Total Weighted Visits, *n*	Share of Overall Cutaneous Wound Visits, %
1	General/family practice	1,718,624.00	132,834.82	1,311,433.61	1,409,196.02	849,732.70	5,421,821.15	27.80%
2	Other specialties	1,764,286.00	1,046.70	1,657,506.19	139,694.19	1,381,919.93	4,944,453.00	25.35%
3	Internal medicine	1,264,406.00	13,627.50	743,398.76	785,173.97	309,684.02	3,116,290.26	15.98%
4	Orthopedic surgery	396,662.00	691,522.65	259,702.76	292,786.35	292,786.35	1,933,460.10	9.91%
5	Pediatrics	416,884.00	26,549.53	311,638.35	379,321.98	712,552.48	1,846,946.35	9.47%
6	General surgery	275,847.00	225.76	225,719.35	203,210.50	59,236.96	764,239.58	3.92%
7	Dermatology	226,153.00	N/A	128,865.83	189,365.91	206,585.90	750,970.64	3.85%
8	Otolaryngology	59,338.00	N/A	21,319.49	39,115.72	135,624.95	255,398.16	1.31%
9	Obstetrics and gynecology	94,667.00	N/A	103,653.31	N/A	17,147.10	215,467.41	1.10%
10	Cardiovascular diseases	N/A	N/A	23,223.13	N/A	41,761.59	64,984.72	0.33%
11	Physician assistant	N/A	50,965.06	N/A	N/A	N/A	50,965.06	0.26%
12	Nurse practitioner	N/A	43,081.24	N/A	N/A	N/A	43,081.24	0.22%
13	Neurology	15,100.00	N/A	N/A	5201.90	16,053.77	36,355.67	0.19%
14	Oncology	24,042.00	N/A	N/A	N/A	N/A	24,042.00	0.12%
15	Urology	N/A	N/A	N/A	23,440.50	N/A	23,440.50	0.12%
16	Ophthalmology	N/A	N/A	N/A	N/A	13,256.78	13,256.78	0.07%
	Total	6,256,009.00	959,853.26	4,786,460.80	3,466,507.04	4,036,342.52	19,505,172.62	100.00%
**Acute Cutaneous Wounds**								
	**Specialty\Year**	**2011**	**2013**	**2014**	**2015**	**2016**	**Total**	**Percentage (%)**
1	General/family practice	1,382,606.00	60,420.86	970,437.11	1,093,521.76	571,063.72	4,078,049.44	31.31%
2	Other specialties	1,101,298.00	720.28	1,114,417.911	79,482.5017	740,587.0978	3,036,505.79	23.32%
3	Pediatrics	363,898.00	26,288.55	290,607.5727	379,321.9844	712,552.4754	1,772,668.58	13.61%
4	Internal medicine	890,684.00	9648.23	277,292.212	428,519.2243	166,312.8063	1,772,456.48	13.61%
5	Orthopedic surgery	335,914.00	N/A	672,870.3446	164,724.225	116,783.7254	1,290,292.29	9.91%
6	General surgery	119,286.00	225.76	67,464.3892	107,471.8797	29,618.47964	324,066.51	2.49%
7	Dermatology	159,192.00	N/A	6668.9907	34,282.6559	106,412.1551	306,555.80	2.35%
8	Otolaryngology	59,338.00	N/A	21,319.4878	37,670.5482	135,624.9503	253,952.99	1.95%
9	Obstetrics and gynecology	55,409.00	N/A	N/A	N/A	17,147.10065	72,556.10	0.56%
10	Physician assistant	N/A	37,467.09	N/A	N/A	N/A	37,467.09	0.29%
11	Nurse practitioner	N/A	30,066.31	N/A	N/A	N/A	30,066.31	0.23%
12	Neurology	N/A	N/A	N/A	N/A	16,053.77028	16,053.77	0.12%
13	Urology	N/A	N/A	N/A	13,580.2053	N/A	13,580.21	0.10%
14	Ophthalmology	N/A	N/A	N/A	N/A	13,256.77797	13,256.78	0.10%
15	Oncology	5402.00	N/A	N/A	N/A	N/A	5402.00	0.04%
	Total	4,473,027.00	164,837.09	3,421,078.02	2,338,574.98	2,625,413.06	13,022,930.14	100%
**Chronic Cutaneous Wounds**								
	**Specialty\Year**	**2011**	**2013**	**2014**	**2015**	**2016**	**Total**	**Percentage (%)**
1	Other specialties	743,081.00	326.41	543,088.28	80,699.26	759,368.56	2,126,563.51	32.79%
2	General/family practice	336,018.00	72,413.96	340,996.51	339,204.70	278,668.98	1,367,302.14	21.08%
3	Internal medicine	373,722.00	3979.27	466,106.55	356,654.75	143,371.21	1,343,833.78	20.72%
4	Dermatology	66,961.00	N/A	122,196.84	155,083.25	134,768.24	479,009.34	7.39%
5	General surgery	156,561.00	N/A	158,254.97	99,225.66	29,618.48	443,660.10	6.84%
6	Orthopedic surgery	60,748.00	N/A	34,993.83	94,978.53	176,002.62	366,722.98	5.65%
7	Obstetrics and gynecology	39,258.00	N/A	103,653.31	N/A	N/A	142,911.31	2.20%
8	Pediatrics	52,986.00	260.99	21,030.78	N/A	N/A	74,277.77	1.15%
9	Cardiovascular diseases	N/A	N/A	23,223.13	N/A	41,761.59	64,984.72	1.00%
10	Neurology	15,100.00	N/A	N/A	5,201.90	N/A	20,301.90	0.31%
11	Oncology	18,640.00	N/A	N/A	N/A	N/A	18,640.00	0.29%
12	Physician assistant	N/A	13,497.97	N/A	N/A	N/A	13,497.97	0.21%
13	Nurse practitioner	N/A	13,014.93	N/A	N/A	N/A	13,014.93	0.20%
14	Urology	9860.30	N/A	N/A	N/A	N/A	9860.30	0.15%
15	Otolaryngology	N/A	N/A	N/A	1445.17	N/A	1445.17	0.02%
	Total	1,874,946.30	105,506.52	1,815,558.20	1,134,508.22	1,565,575.68	6,486,025.92	100.00%

Estimates are restricted to survey years with detailed specialty information (2011 and 2013–2016).

**Table 2:** Distribution of acute, chronic, and overall cutaneous wound visits across primary care, surgical, and medical specialties by survey year, NAMCS 2011–2019.

	Acute Wound Visits			Chronic Wound Visits			Overall Wound Visits		
Survey Year	Primary Care, Weighted Visits, *n*	Surgical Specialties, Weighted Visits, *n*	Medical Specialties, Weighted Visits, *n*	Primary Care, Weighted Visits, *n*	Surgical Specialties, Weighted Visits, *n*	Medical Specialties, Weighted Visits, *n*	Primary Care, Weighted Visits, *n*	Surgical Specialties, Weighted Visits, *n*	Medical Specialties, Weighted Visits, *n*
2011	2,692,597.00	1,184,394.00	596,036.00	801,984.00	815,241.00	245,850.00	3,494,581.00	1,919,542.00	841,886.00
2012	133,733.44	N/A	1547.85	104,907.26	N/A	N/A	238,640.69	N/A	1547.85
2013	96,357.64	225.76	720.28	76,654.21	N/A	326.41	173,011.85	225.76	1046.70
2014	1,538,336.89	1,432,769.85	449,971.27	929,590.79	616,820.54	267,132.87	2,467,927.68	2,033,248.87	717,104.14
2015	1,901,362.96	359,737.38	77,474.63	695,859.45	205,509.66	240,984.41	2,573,691.97	561,760.00	297,971.47
2016	1,467,076.10	425,227.05	733,109.91	422,040.19	728,918.20	412,601.30	1,889,116.29	1,154,145.24	993,080.98
2018	1,339,458.91	59,499.19	524,233.27	219,853.69	542,691.08	918,325.98	1,559,312.60	602,190.27	1,312,369.29
2019	3,324,498.05	3,448,465.40	6,934,784.03	2,528,602.23	460,122.43	21,192.02	5,853,100.27	3,908,587.83	6,955,976.06
Total	12,493,420.99	6,910,318.63	9,317,877.26	5,779,491.81	3,369,302.90	2,106,413.00	18,249,382.36	10,179,699.98	11,120,982.48

Estimates are based on the NAMCS physician category variable (primary care, surgical specialties, medical specialties), available in the public-use files for 2011–2019. N/A indicates years in which the physician category variable was not available or did not support stable estimates for that stratum.

**Table 3:** Top 20 antimicrobial medications at cutaneous wound visits overall and by wound type, NAMCS 2011–2019.

	Overall Wound Visits				Acute Wound Visits				Chronic Wound Visits			
Rank	Medication	Weighted Frequency, n	% of All Medications at Overall Wound Visits	% of Antimicrobial Medications at Overall Wound Visits	Medication	Weighted Frequency, n	% of All Medications at Acute Wound Visits	% of Antimicrobial Medications at Acute Wound Visits	Medication	Weighted Frequency, n	% of All Medications at Chronic Wound Visits	% of Antimicrobial Medications at Chronic Wound Visits
1	Cephalexin	4,385,163.88	4.22%	32.15%	Cephalexin	4,083,971.40	5.84%	39.22%	Topical Mupirocin	938,338.58	1.43%	14.02%
2	Topical Mupirocin	1,650,607.54	1.59%	12.10%	Miscellaneous Antibiotics	1,137,657.10	1.63%	10.92%	Cephalexin	798,112.92	1.21%	11.92%
3	Miscellaneous Antibiotics	1,229,227.37	1.18%	9.01%	Topical Silver Sulfadiazine	1,098,971.48	1.57%	10.55%	Sulfamethoxazole-Trimethoprim	668,067.60	1.02%	9.98%
4	Topical Silver Sulfadiazine	1,037,339.55	1.00%	7.60%	Fluconazole	267,968.96	0.38%	2.57%	Topical Nystatin	495,624.81	0.75%	7.40%
5	Sulfamethoxazole-Trimethoprim	833,900.19	0.80%	6.11%	Topical Mupirocin	820,146.48	1.17%	7.88%	Ciprofloxacin	471,428.86	0.72%	7.04%
6	Amoxicillin-Clavulanate	715,430.86	0.69%	5.24%	Bacitracin	487,216.16	0.70%	4.68%	Topical Chlorhexidine	424,299.90	0.64%	6.34%
7	Topical Chlorhexidine	482,578.05	0.46%	3.54%	Ciprofloxacin	411,797.75	0.59%	3.95%	Topical Silver Sulfadiazine	391,280.50	0.59%	5.84%
8	Ciprofloxacin	480,669.44	0.46%	3.52%	Amoxicillin-Clavulanate	342,954.36	0.49%	3.29%	Amoxicillin-Clavulanate	385,496.63	0.59%	5.76%
9	Bacitracin	448,190.88	0.43%	3.29%	Sulfamethoxazole-Trimethoprim	291,540.58	0.42%	2.80%	Fluconazole	321,318.43	0.49%	4.80%
10	Clindamycin	374,606.86	0.36%	2.75%	Gentamicin	227,102.37	0.32%	2.18%	Azithromycin	228,908.84	0.35%	3.42%
11	Levofloxacin	323,986.31	0.31%	2.38%	Clindamycin	214,847.64	0.31%	2.06%	Hydroxychloroquine	211,287.81	0.32%	3.16%
12	Doxycycline	308,333.41	0.30%	2.26%	Topical Chlorhexidine	207,636.34	0.30%	1.99%	Clindamycin	177,367.06	0.27%	2.65%
13	Fluconazole	267,968.96	0.26%	1.96%	Cefdinir	201,709.11	0.29%	1.94%	Levofloxacin	174,004.66	0.26%	2.60%
14	Gentamicin	227,102.37	0.22%	1.66%	Levofloxacin	170,093.46	0.24%	1.63%	Doxycycline	170,196.83	0.26%	2.54%
15	Cefdinir	201,709.11	0.19%	1.48%	Doxycycline	145,957.38	0.21%	1.40%	Ketoconazole	163,969.94	0.25%	2.45%
16	Topical Silver Nitrate	165,729.00	0.16%	1.21%	Minocycline	73,994.76	0.11%	0.71%	Topical Clotrimazole	153,451.93	0.23%	2.29%
17	Topical Clotrimazole	144,256.98	0.14%	1.06%	Cefadroxil	59,674.00	0.09%	0.57%	Piperacillin-Tazobactam	147,225.00	0.22%	2.20%
18	Metronidazole	127,211.29	0.12%	0.93%	Neomycin-Polymyxin B Sulfate	62,124.78	0.09%	0.60%	Topical Silver Nitrate	137,415.27	0.21%	2.05%
19	Ceftriaxone	118,837.22	0.11%	0.87%	Metronidazole	60,188.00	0.09%	0.58%	Ceftriaxone	118,483.21	0.18%	1.77%
20	Cefuroxime	118,035.73	0.11%	0.87%	Cefazolin	48,336.32	0.07%	0.46%	Cefuroxime	118,035.73	0.18%	1.76%
	Total	13,640,885.01	13.13%	100.00%	Total	10,413,888.41	14.88%	100.00%	Total	6,694,314.50	10.18%	100.00%

## Data Availability

NAMCS public-use data files and documentation for 2011–2019 are available from the National Center for Health Statistics website (Centers for Disease Control and Prevention).

## Supplementary Materials

Supporting information can be downloaded at: https://www.mdpi.com/article/10.3390/antibiotics15020142/s1. Figure S1. Top antimicrobial medications across wound types (NAMCS, 2011–2019). Bars show the weighted share (%) among antimicrobial medications recorded at outpatient cutaneous wound visits, overall and stratified by acute (open/traumatic wounds and burns) and chronic (pressure injuries and lower-limb ulcers) wounds. Estimates incorporate NAMCS survey design (weights, strata, primary sampling units); values reflect composition among antimicrobials, not per-visit antibiotic rates.; Figure S2. Antimicrobial prescribing patterns overview (NAMCS, 2011–2019). Top panel compares the top five agents by wound type (weighted composition among antimicrobial medications). Bottom-left panel shows systemic vs topical composition among antimicrobial medications (overall, acute, chronic). Bottom-right list highlights wound-type–dominant agents. Survey design features were applied; composition measure, not per-visit rates.; Figure S3. (A–C) Top 15 antimicrobial agents by wound type (NAMCS, 2011–2019). Bars display the weighted share (%) among antimicrobial medications for (A) overall cutaneous wounds, (B) acute wounds, and (C) chronic wounds. Estimates account for survey weights/strata/PSUs; percentages may not total 100% due to rounding. “Miscellaneous antibiotics” reflects nonspecific antimicrobial codes in NAMCS.; Table S1. Top 20 cutaneous wound diagnoses overall and by wound type in US office-based visits, NAMCS 2011–2019.; Table S2. Prescription status of medications at cutaneous wound visits overall and by wound type, NAMCS 2011–2019.; Table S3. *p*-values for linear trends over time in antimicrobial share among medications at cutaneous wound visits, by wound type, NAMCS 2011–2019.; Table S4. *p*-values for linear trends over time in the share of cutaneous wound visits by specialty category, NAMCS 2011–2019.

## Abbreviations

COVID-19	Coronavirus disease 2019
ICD-9-CM	International Classification of Diseases, Ninth Revision, Clinical Modification
ICD-10-CM	International Classification of Diseases, Tenth Revision, Clinical Modification
NAMCS	National Ambulatory Medical Care Survey
SAS	Statistical Analysis System
