# Supplementary material for: Outpatient Cutaneous Wound Care in the United States: Specialty Distribution and Antimicrobial Prescribing Patterns
Source: Antibiotics (Basel). 2026 Feb 1;15(2):142. doi: 10.3390/antibiotics15020142 (PMC12937199; doi:10.3390/antibiotics15020142)

Supplementary Figures

Figure S1. Top antimicrobial medications across wound types (NAMCS, 2011–2019). Bars show the weighted share (%) among antimicrobial medications recorded at outpatient cutaneous wound visits, overall and stratified by acute (open/traumatic wounds and burns) and chronic (pressure injuries and lower-limb ulcers) wounds. Estimates incorporate NAMCS survey design (weights, strata, primary sampling units); values reflect composition among antimicrobials, not per-visit antibiotic rates.

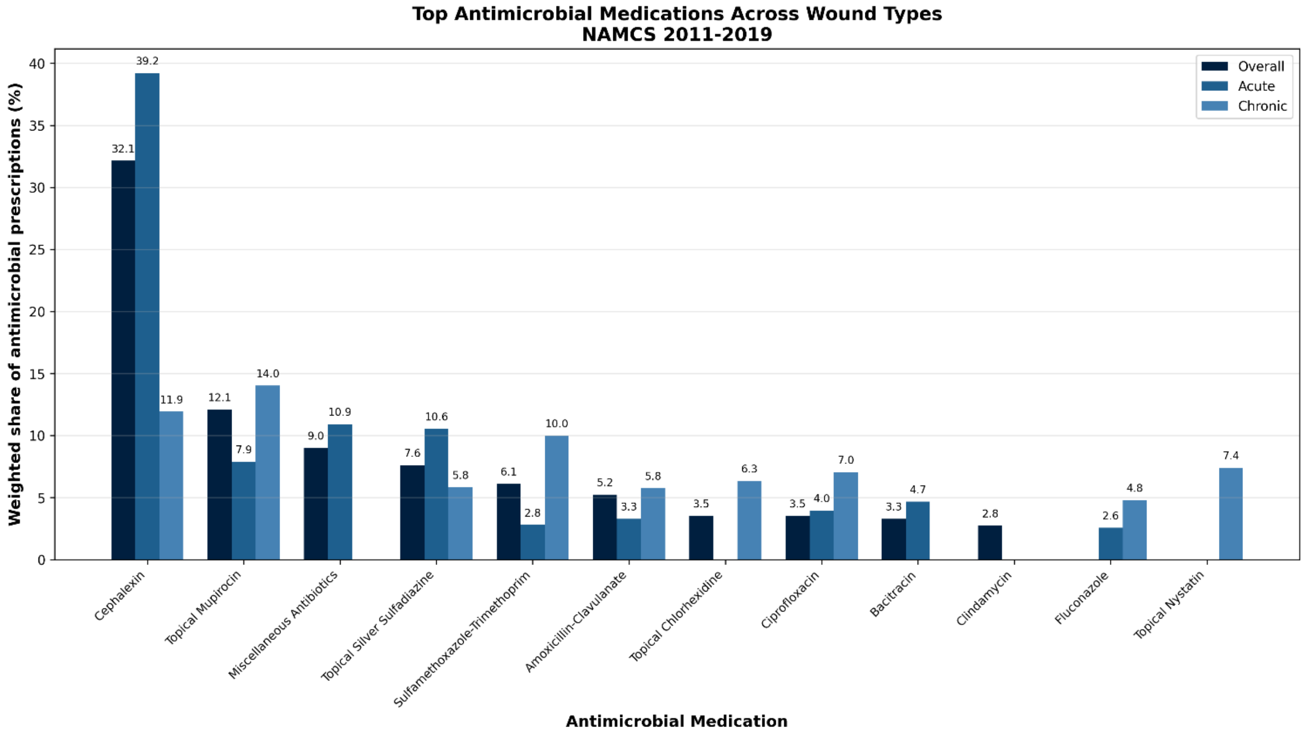

Figure S2. Antimicrobial prescribing patterns overview (NAMCS, 2011–2019). Top panel compares the top five agents by wound type (weighted composition among antimicrobial medications). Bottom-left panel shows systemic vs topical composition among antimicrobial medications (overall, acute, chronic). Bottom-right list highlights wound-type–dominant agents. Survey design features were applied; composition measure, not per-visit rates.

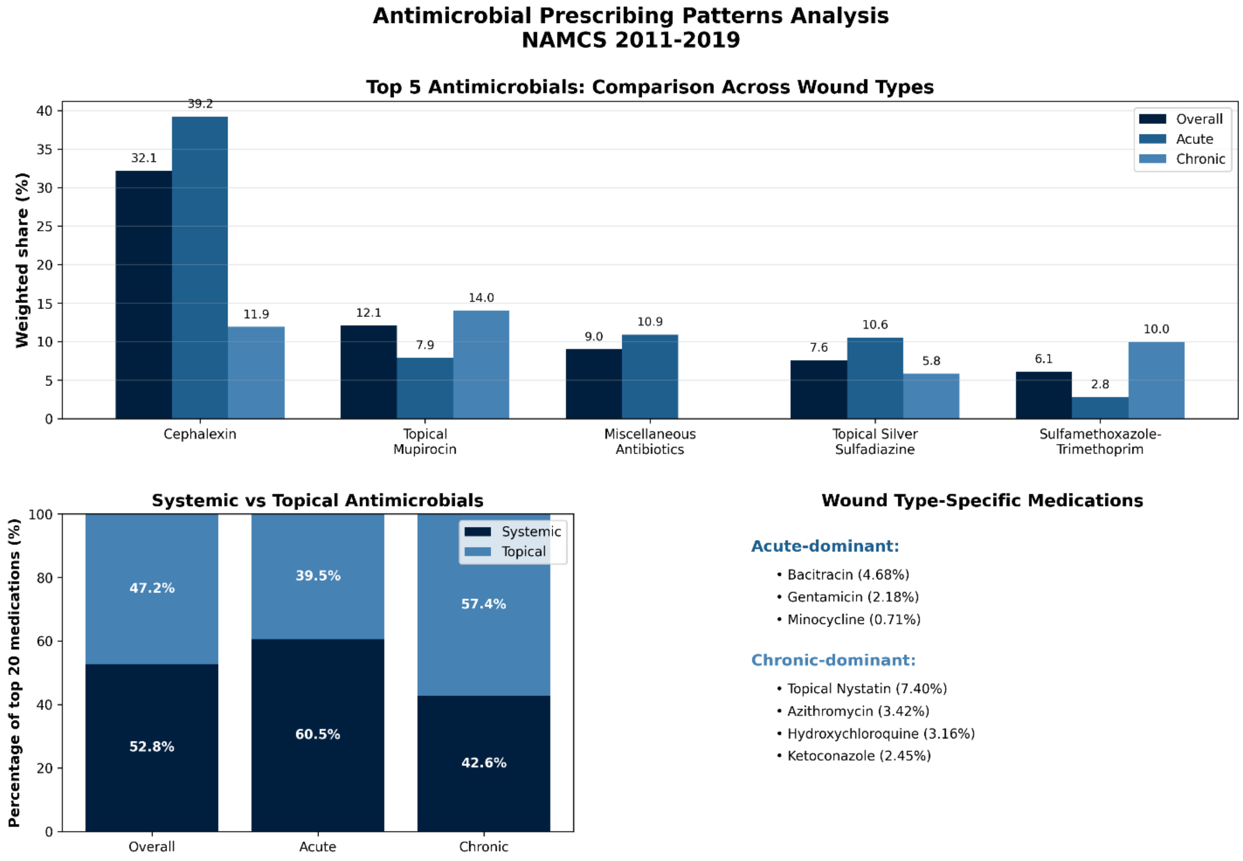

Note: Wound type-specific medications were identified by comparing the top 20 antimicrobials prescribed across wound types. "Acute-dominant" indicates medications that ranked in the top 20 for acute cutaneous wounds but were absent from the chronic wounds top 20 list. "Chronic-dominant" indicates medications that ranked in the top 20 for chronic cutaneous wounds but were absent from the acute wounds top 20 list. The presence of antifungals (Nystatin, Ketoconazole) and immunomodulators (Hydroxychloroquine) among chronic-dominant medications suggests different microbial profiles and treatment approaches for chronic wounds. Data derived from weighted prescription frequencies in NAMCS 2011-2019.

Figure S3. (A–C) Top 15 antimicrobial agents by wound type (NAMCS, 2011–2019). Bars display the weighted share (%) among antimicrobial medications for (A) overall cutaneous wounds, (B) acute wounds, and (C) chronic wounds. Estimates account for survey weights/strata/PSUs; percentages may not total 100% due to rounding. “Miscellaneous antibiotics” reflects nonspecific antimicrobial codes in NAMCS.

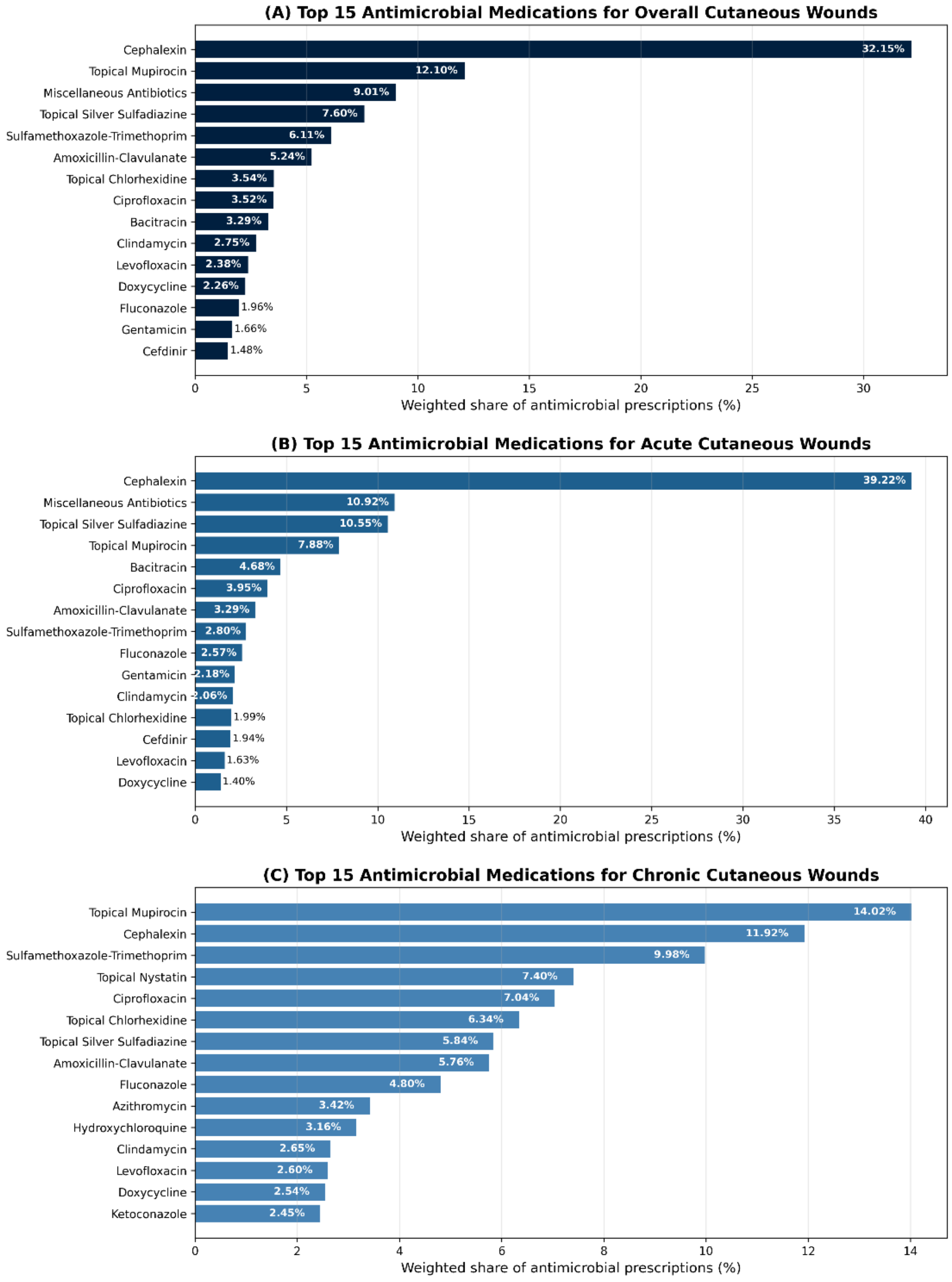

Supplement: Supplementary file 1 [file antibiotics-15-00142-s001.zip › Supplementary Files/Supplementary Figures.pdf]
