# Supplementary material for: Outpatient Cutaneous Wound Care in the United States: Specialty Distribution and Antimicrobial Prescribing Patterns
Source: Antibiotics (Basel). 2026 Feb 1;15(2):142. doi: 10.3390/antibiotics15020142 (PMC12937199; doi:10.3390/antibiotics15020142)
Supplement: Supplementary file 1 [file antibiotics-15-00142-s001.zip › Supplementary Files/Supplementary Methods.pdf]

Supplementary Methods

S1. Data Source and Study Design

We performed a cross-sectional analysis of the National Ambulatory Medical Care Survey (NAMCS) public-use files from 2011 to 2019. NAMCS is a nationally representative survey of visits to non-federal, office-based physicians in the United States conducted by the National Center for Health Statistics (NCHS), Centers for Disease Control and Prevention. NAMCS uses a multistage probability design that samples geographic primary sampling units (PSUs), physician practices within PSUs, and patient visits within sampled practices.

For each sampled visit, NAMCS provides a visit weight, stratum identifier, and PSU indicator that account for differential probabilities of selection, nonresponse, and post-stratification adjustments. When these design variables are incorporated into survey procedures, estimates can be extrapolated to national visit counts and proportions.

We restricted our analysis to visits in the NAMCS physician component, which includes office-based physicians providing direct patient care. Visits to hospital-based outpatient clinics, emergency departments, and settings where nonphysician clinicians are the primary providers are not captured in the physician component and were not included in this analysis.

S2. Case Definitions and Wound Classification

Diagnoses in NAMCS are recorded using the International Classification of Diseases, Ninth Revision, Clinical Modification (ICD-9-CM) through September 30, 2015, and the International Classification of Diseases, Tenth Revision, Clinical Modification (ICD-10-CM) beginning October 1, 2015. In earlier years, up to three diagnosis fields are available per visit; in later years, up to five fields are available.

Cutaneous wound visits were identified by scanning all available diagnosis fields for codes representing an acute or chronic break in skin integrity. We developed prespecified lists of ICD-9-CM and ICD-10-CM codes, grouped into acute and chronic wound categories based on wound type, anatomic location, and chronicity. The detailed code enumeration is provided in Supplementary Table S1 and is based on the classification scheme summarized in the NAMCS wound-code specification file.

Acute cutaneous wounds comprised open or traumatic wounds of the head, neck, trunk, and extremities and burns of the skin and extremities. Anchor code families for acute wounds included:

- ICD-10-CM S01–S91- (open wounds of head and neck, trunk, and extremities)
- ICD-9-CM 870–894 (open wounds by anatomic site)
- ICD-10-CM T20–T31 (burns of external body surface and extremities)
- ICD-9-CM 945–949 (burns of lower limb and multiple/unspecified sites)

Chronic cutaneous wounds comprised pressure injuries and non-pressure lower-limb ulcers (including venous and diabetic patterns) as well as selected genital and perineal ulcers when clearly chronic. Anchor code families for chronic wounds included:

- ICD-10-CM L89- (pressure injuries)
- ICD-10-CM L97- (non-pressure chronic ulcer of lower limb)
- ICD-10-CM I83.0–I83.2 (varicose veins of lower extremities with ulcer, with or without inflammation)
- ICD-9-CM 707.xx (pressure ulcers and other chronic ulcers)

Within these anchor families, individual codes for specific sites and stages (for example, L89.0–L89.9 for staged pressure injuries or 707.1x for lower-extremity ulcers) were included as listed in Supplementary Methods Table S1.

For each visit, we scanned all available diagnosis fields in that survey year. Visits with at least one acute wound code anywhere in the diagnosis fields were classified as acute wound visits. Visits with at least one chronic wound code were classified as chronic wound visits. Encounters that contained both acute and chronic wound codes were flagged and counted once in overall wound analyses; in sensitivity checks, these encounters were grouped with chronic wounds because chronic wounds typically dominate long-term management needs.

S3. Medication Data and Antimicrobial Classification

NAMCS records medications that are prescribed, provided, or continued at each sampled visit. In 2011–2013, up to eight medications per visit were recorded; in subsequent years, up to thirty medications could be listed. Each medication entry includes a drug name, therapeutic class codes, and indicators for prescription versus nonprescription status.

We abstracted all recorded medications for visits that met our cutaneous wound

definitions. Medications were treated as individual drug mentions rather than visit-level indicators. Analyses of medication patterns therefore describe the distribution of drug mentions among medications recorded at wound visits, not the proportion of visits with at least one drug in a given class.

Antimicrobial medications were defined a priori as systemic or topical antibiotics, antifungals, or antivirals. We used NAMCS therapeutic class codes, generic names, and route information to identify and classify antimicrobials. Topical antiseptics (for example, chlorhexidine and povidone–iodine), dressings, advanced wound-care products, biologic agents, and other non-antimicrobial therapies were not counted as antimicrobials in this analysis. Antimicrobials were categorized by route of administration as systemic (oral, parenteral, or other non-topical formulations) or topical (creams, ointments, gels, solutions, and related dermal preparations).

Within antimicrobials, we further categorized agents into three main classes: antibiotics, antifungals, and antivirals. Among antibiotics, we identified key individual agents of interest, including cephalexin, amoxicillin–clavulanate, clindamycin, trimethoprim–sulfamethoxazole, and doxycycline. Among topical agents, mupirocin, topical nystatin, and topical azole antifungals were flagged given their frequent use in cutaneous wound care. Antifungals and antivirals were included when reporting overall antimicrobial composition but were not counted as antibiotics in antibiotic-specific summaries. Mapping of specific drug names and therapeutic classes to antimicrobial categories is detailed in Supplementary Methods Table S2.

Prescription status was summarized using NAMCS indicators, which distinguish prescription-only drugs from drugs that are available either by prescription or over-the-counter and those that are over-the-counter only.

S4. Physician Specialty and Provider Categories

Physician specialty in NAMCS is coded using three-digit specialty identifiers derived from the American Medical Association classification. In the public-use files, detailed specialty codes are available for selected years; for this analysis, we used detailed specialty identifiers for wound visits in 2011 and 2013–2016. Using these detailed codes, we described the distribution of wound visits across individual specialties, including general or family practice, internal medicine, pediatrics, orthopedic surgery, general surgery, dermatology, otolaryngology, obstetrics and gynecology, cardiovascular disease, and other subspecialties. Because dermatology is of particular interest, dermatology-specific estimates were reported when detailed specialty codes were available.

To provide a consistent specialty framework across the entire 2011–2019 period, we also used the NAMCS physician category variable, which groups individual specialties into broader categories: primary care, surgical specialties, and medical specialties. In this classification, general or family practice, internal medicine, and pediatrics were grouped as primary care. Orthopedic surgery, general surgery, otolaryngology, obstetrics and gynecology, urology, and related fields were grouped as surgical specialties. Dermatology and other internal medicine subspecialties (for example, cardiology, endocrinology, rheumatology, nephrology, pulmonology, gastroenterology, allergy/immunology, geriatrics, and physical medicine and rehabilitation) were grouped as medical specialties.

The mapping of three-digit specialty codes to the primary care, surgical, and medical specialty categories is summarized in Supplementary Methods Table S3.

S5. Outcomes and Statistical Analysis

The primary outcomes of interest were: (1) nationally weighted counts and proportions of acute, chronic, and overall cutaneous wound visits among all office-based physician visits; (2) the distribution of wound visits across individual specialties (for years with detailed specialty codes) and across primary care, surgical, and medical physician categories for all years; and (3) the proportion and composition of antimicrobial medications among all medications recorded at wound visits, overall and stratified by wound type.

We used design-based survey procedures that incorporate the NAMCS visit weights, strata, and PSUs to generate national estimates of visit counts, medication counts, proportions, and 95% confidence intervals. For time-trend analyses, we computed annual weighted estimates for key outcomes and assessed linear trends over survey years by fitting survey-weighted linear regression models with calendar year entered as a continuous predictor.

Two-sided p values for trend were obtained from the coefficient for calendar year in the survey-weighted regression models. We considered $p < 0.05$ statistically significant. When reporting p values, we focused on the presence or absence of monotonic linear trends over time rather than on effect sizes alone.

Following NCHS guidance on data reliability for NAMCS, we considered estimates potentially unreliable when based on fewer than 30 unweighted observations or when the relative standard error exceeded 30%. Such estimates were suppressed in tables or combined with adjacent categories as appropriate. Where suppression or aggregation was applied, this is noted in the corresponding table footnotes.

All data management and analyses were conducted using SAS version 9.4 (SAS Institute Inc., Cary, North Carolina) with survey procedures (for example, PROC SURVEYMEANS, PROC SURVEYFREQ, and PROC SURVEYREG). Visit-based outcomes treated the visit as the unit of analysis, whereas medication-based outcomes treated the individual drug mention as the unit of analysis.

S6. Ethics

NAMCS public-use files contain de-identified data. The NCHS Ethics Review Board provides oversight for the NAMCS program under a standing approval protocol. This secondary analysis of public-use data was considered exempt from additional institutional review board review.

Supplementary Methods Table S1. ICD-9-CM and ICD-10-CM code families used to define acute and chronic cutaneous wounds

Wound category	Clinical description	ICD-10-CM code families	ICD-9-CM code families
Acute open or traumatic wounds of head and neck, trunk, and extremities	Lacerations and open wounds of scalp, face, trunk, upper extremity, and lower extremity	S01–S09, S11–S19, S21–S29, S31–S39, S41–S49, S51–S59, S61–S69, S71–S79, S81–S89, S91–S99	870–879, 881–894
Other acute open wounds of trunk and external genitalia	Open wounds of chest wall, abdominal wall, perineum, and external genital organs	S31.0–S31.9, S35–S39, S31.5–S31.8	879.2–879.5, 878.0–878.9
Acute burns of skin and extremities	Burns and corrosions of external body surface and extremities	T20–T25, T30–T31	945–949, 948.0–948.9
Pressure injuries	Pressure injuries by site and stage	L89.x	707.0x, 707.2x
Non-pressure chronic ulcers of lower limb	Chronic ulcer of leg and foot, including venous, arterial, and other lower-limb ulcers	L97.x	707.1x
Varicose veins of lower extremity with ulcer	Varicose veins of lower extremity with ulcer, with or without inflammation	I83.0–I83.2	454.0, 454.2, 454.8

Other chronic ulcers and related chronic wounds	Other specified chronic skin ulcers, including heel, mid-foot, and ankle ulcers not otherwise classified	L98.4, L98.8 (when explicitly chronic), selected N48/N76 codes as specified in code list	707.8–707.9, selected 707.3x, 707.9x as specified in code list

Note: This table summarizes the main ICD-9-CM and ICD-10-CM code families used to define acute and chronic cutaneous wounds in NAMCS. A full enumerated list of individual diagnosis codes, including specific fourth- and fifth-digit extensions, is provided in the internal wound-code specification file that underlies these groupings.

Supplementary Methods Table S2. Mapping of medications to antimicrobial categories

Antimicrobial class	Route category	Included example agents	Excluded agents in related classes
Systemic antibiotics	Systemic (oral, parenteral, other non-topical)	Cephalexin; amoxicillin–clavulanate; clindamycin; trimethoprim–sulfamethoxazole; doxycycline; ciprofloxacin; other systemic antibacterial agents coded in NAMCS	Topical antibiotics; topical antiseptics (e.g., chlorhexidine, povidone–iodine); non-antibacterial wound products
Topical antibiotics	Topical (cream, ointment, gel, solution, other dermal)	Mupirocin; neomycin-containing combinations; bacitracin; polymyxin B-containing combinations; other topical antibacterials	Systemic antibiotics; topical antiseptics; silver dressings; advanced wound dressings
Systemic antifungals	Systemic	Fluconazole; itraconazole; terbinafine (oral); other systemic antifungals coded in NAMCS	Topical antifungals; nystatin oral suspensions used for mucosal disease
Topical antifungals	Topical	Topical nystatin; topical azoles (clotrimazole, miconazole, ketoconazole);	Systemic antifungals; topical antiseptics

		ciclopirox; other topical antifungals	
Systemic antivirals	Systemic	Acyclovir; valacyclovir; famciclovir; other systemic antivirals coded in NAMCS	Topical antivirals; ophthalmic antivirals
Topical antivirals	Topical	Topical acyclovir; penciclovir; other topical antivirals when present	Systemic antivirals; ophthalmic antivirals; topical antiseptics

Note: Antimicrobials were identified using NAMCS therapeutic class codes, generic names, and route information. Topical antiseptics, wound dressings, advanced wound products, and biologic agents were excluded from antimicrobial counts even when used in wound care.

Supplementary Methods Table S3. Mapping of NAMCS physician specialties to analytic specialty categories

Analytic specialty category	Included NAMCS physician specialties (examples)	Comments
Primary care	General or family practice; internal medicine; pediatrics	Includes office-based adult and pediatric primary care physicians.
Surgical specialties	Orthopedic surgery; general surgery; otolaryngology (ENT); obstetrics and gynecology; urology; other surgical subspecialties	Includes office-based surgeons who may manage traumatic wounds and perioperative wound care.
Medical specialties	Dermatology; cardiology; endocrinology; rheumatology; nephrology; pulmonology; gastroenterology; allergy/immunology; geriatrics; physical medicine and rehabilitation; other internal medicine subspecialties	Dermatology is counted within medical specialties in NAMCS. Dermatology-specific estimates were also reported separately where detailed codes were available.

Note: Mapping is based on the NAMCS three-digit physician specialty codes and the corresponding physician category variable (SPECCAT). Some low-frequency specialties were combined into the most appropriate analytic category to preserve estimate stability.
