# Supplementary material for: Outpatient Cutaneous Wound Care in the United States: Specialty Distribution and Antimicrobial Prescribing Patterns
Source: Antibiotics (Basel). 2026 Feb 1;15(2):142. doi: 10.3390/antibiotics15020142 (PMC12937199; doi:10.3390/antibiotics15020142)
Supplement: Supplementary file 1 [file antibiotics-15-00142-s001.zip › Supplementary Files/Supplementary Tables.pdf]

Supplementary Data Tables

Supplementary Table S1. Top 20 cutaneous wound diagnoses overall and by wound type in US office-based visits, NAMCS 2011–2019.

		Overall wound visits			Acute wound visits			Chronic wound visits		
Rank	Diagnosis	Weighted visits, n	% of all NAMCS visits	% of all cutaneous wound diagnoses	Diagnosis	Weighted visits, n	% of acute cutaneous wound diagnoses	Diagnosis	Weighted visits, n	% of chronic cutaneous wound diagnoses
1	Open wound of thumb without damage to nail	3,587,405.14	0.06%	7.96%	Open wound of thumb without damage to nail	3,587,405.14	10.88%	Non-pressure chronic ulcer of other part of foot	2,287,878.47	17.78%
2	Open wound of lower leg	2,593,611.91	0.05%	5.75%	Open wound of lower leg	2,593,611.91	7.87%	Non-pressure chronic ulcer of skin, not elsewhere classified	1,206,654.24	9.38%
3	Non-pressure chronic ulcer of other part of foot	2,287,878.47	0.04%	5.08%	Open wound of ear	2,255,805.04	6.84%	Ulcer of lower limbs, excluding decubitus, unspecified	1,121,576.95	8.72%
4	Open wound of ear	2,255,805.04	0.04%	5.00%	Open wound of finger(s), uncomplicated	1,730,254.11	5.25%	Ulcer of other part of foot, excluding decubitus	989,776.32	7.69%
5	Open wound of finger(s), uncomplicated	1,730,254.11	0.03%	3.84%	Open wound of foot	1,596,539.82	4.84%	Non-pressure chronic ulcer of unspecified part of lower leg	793,095.06	6.16%
6	Open wound of foot	1,596,539.82	0.03%	3.54%	Open wound of knee/leg [except thigh]/ankle, uncomplicated	1,383,761.86	4.20%	Non-pressure chronic ulcer of lower limb, not elsewhere classified	734,747.09	5.71%
7	Open wound of knee/leg [except thigh]/ankle, uncomplicated	1,383,761.86	0.02%	3.07%	Open wound of upper arm	1,379,195.06	4.18%	Chronic ulcer of unspecified site	576,711.44	4.48%
8	Open wound of upper arm	1,379,195.06	0.02%	3.06%	Open wound of knee	1,230,939.61	3.73%	Varicose veins of lower extremities with ulcer	535,811.93	4.16%
9	Open wound of knee	1,230,939.61	0.02%	2.73%	Open wound of ankle	1,213,442.44	3.68%	Pressure ulcer of buttock	456,671.31	3.55%
10	Open wound of ankle	1,213,442.44	0.02%	2.69%	Burn [any degree] involving less than 10% body surface	921,959.55	2.80%	Pressure ulcer, unspecified stage	391,613.58	3.04%
11	Non-pressure chronic ulcer of skin, not	1,206,654.24	0.02%	2.68%	Open wound of other finger	879,469.92	2.67%	Chronic ulcer of other	355,571.52	2.76%

	elsewhere classified				without damage to nail			specified sites		
12	Ulcer of lower limbs, exc decubitus, unspecified	1,121,576.95	0.02%	2.49%	Open wound foot except toe(s) alone with tendon involvement	863,364.43	2.62%	Ulcer of penis	336,049.14	2.61%
13	Ulcer of other part of foot, excluding decubitus	989,776.32	0.02%	2.20%	Open wound of hand	859,264.70	2.61%	Pressure ulcer of unspecified site	295,878.54	2.30%
14	Burn [any degree] involving less than 10% body surface	921,959.55	0.02%	2.05%	Open wound of hand except finger(s) alone, uncomplicated	655,658.95	1.99%	Decubitus ulcer of unspecified site	264,660.74	2.06%
15	Open wound of other finger without damage to nail	879,469.92	0.02%	1.95%	Burn of second degree of wrist and hand	572,990.22	1.74%	Ulceration of vulva	247,532.10	1.92%
16	Open wound foot except toe(s) alone with tendon involvement	863,364.43	0.02%	1.92%	Open wound of knee/leg [except thigh]/ankle, complicated	440,942.80	1.34%	Decubitus ulcer of other site	222,160.70	1.73%
17	Open wound of hand	859,264.70	0.02%	1.91%	Open wound of toe with damage to nail	409,608.56	1.24%	Ulcer other part lower limbs, excluding decubitus	212,192.20	1.65%
18	Non-pressure chronic ulcer of unspecified part of lower leg	793,095.06	0.01%	1.76%	Open wound of scalp, without mention of complication	385,005.01	1.17%	Open wound of lower leg	164,784.46	1.28%
19	Non-pressure chronic ulcer of lower limb, not elsewhere classified	734,747.09	0.01%	1.63%	Open wound of scalp	370,436.65	1.12%	Ulceration of vulva, unspecified	161,775.05	1.26%
20	Open wound of hand except finger(s) alone, uncomplicated	655,658.95	0.01%	1.45%	Burn of unspecified degree of lower limb, except ankle and foot	367,236.53	1.11%	Pressure ulcer	161,194.70	1.25%
	**Total**	**28,284,400.68**	**0.49%**	**62.74%**	**Total**	**23,696,892.32**	**71.89%**	**Total**	**11,516,335.56**	**89.51%**

Supplementary Table S2. Prescription status of medications at cutaneous wound visits overall and by wound type, NAMCS 2011–2019.

	**Overall wound visits**		**Acute wound visits**		**Chronic wound visits**	
**Medication status**	**Weighted Frequency**	**Percentage of Total Medications**	**Weighted Frequency**	**Percentage of Total Medications**	**Weighted Frequency**	**Percentage of Total Medications**
Prescription drug	114,433,606.57	73.08%	67,931,113.94	73.35%	47,864,568.28	72.75%
Both Prescription and Over-The-Counter	34,661,273.27	22.13%	20,471,312.00	22.10%	14,523,171.79	22.07%
Undetermined	4,048,832.01	2.59%	2,445,441.15	2.64%	1,603,390.86	2.44%
Nonprescription drug	3,451,588.11	2.20%	1,769,159.02	1.91%	1,800,464.82	2.74%
**Total**	**156,595,299.96**	**100.00%**	**92,617,026.12**	**100.00%**	**65,791,595.74**	**100.00%**

Supplementary Table S3. p-values for linear trends over time in antimicrobial share among medications at cutaneous wound visits, by wound type, NAMCS 2011–2019.

Medication category	p for trend in overall wound visits	p for trend in acute wound visits	p for trend in acute wound visits
Overall (Antimicrobial)	0.195	0.222	0.120
Antibiotic	0.215	0.256	0.091
Antiviral	0.905	0.701	0.037
Antifungal	0.219	0.243	0.137

Note: Each p-value reflects a test for linear trend over time in the share of medications at cutaneous wound visits that belong to the specified antimicrobial category. For each survey year (2011–2019), we calculated the proportion of all medications recorded at wound visits that were antimicrobials (overall, antibiotics, antifungals, or antivirals), separately for overall, acute, and chronic wound visits. Survey weighted linear regression models with calendar year as a continuous predictor were used to estimate p-value for trend.

Supplementary Table S4. p-values for linear trends over time in the share of cutaneous wound visits by specialty category, NAMCS 2011–2019.

Type of cutaneous wounds	p for trend in share of visits provided by primary care	p for trend in share of visits provided by primary care	p for trend in share of visits provided by medical specialties
Acute	0.315	0.434	0.0887
Chronic	0.195	0.384	0.471
Overall	0.240	0.458	0.056

Note: Each p value reflects a test for linear trend over time in the share of cutaneous wound visits provided by each specialty category. For each survey year (2011–2019), we calculated the proportion of cutaneous wound visits managed by primary care, surgical specialties, and medical specialties within each wound type (acute, chronic, overall). Survey weighted linear regression models with calendar year as a continuous predictor were used to estimate p-value for trend in these specialty shares.
